# Left Handedness

**Published:** 1871-08

**Authors:** 


### LEFT HANDEDNESS.

A left handed nurse will make a left handed child, because the infant's right arm, pressed against the nurse, prevents its ready use. If begun early, the child may be easily broken of the habit by causing it to wear a woolen mitten, which is quite as efficient in breaking up the habit of sucking the thumb.

The tendency to use the right hand instead of the left is doubtless inherited. The earlier races of mankind were warlike, the weapons being the bludgeon and the sword; and perhaps early observation showed that the region of the heart, the left side, being most vulnerable, while the right arm was striking the foe the left arm was free to guard the heart.

